# Modified Gene Editing Systems: Diverse Bioengineering Tools and Crop Improvement

**DOI:** 10.3389/fpls.2022.847169

**Published:** 2022-03-17

**Authors:** Guoning Zhu, Hongliang Zhu

**Affiliations:** College of Food Science and Nutritional Engineering, China Agricultural University, Beijing, China

**Keywords:** modified gene editing system, CRISPR, BEs, PE, genome engineering, transgene-free

## Abstract

Gene-editing systems have emerged as bioengineering tools in recent years. Classical gene-editing systems include zinc-finger nucleases (ZFNs), transcription activator-like effector nucleases (TALENs), and clustered regularly interspaced short palindromic repeats (CRISPR) with CRISPR-associated protein 9 (Cas9) (CRISPR/Cas9), and these tools allow specific sequences to be targeted and edited. Various modified gene-editing systems have been established based on classical gene-editing systems. Base editors (BEs) can accurately carry out base substitution on target sequences, while prime editors (PEs) can replace or insert sequences. CRISPR systems targeting mitochondrial genomes and RNA have also been explored and established. Multiple gene-editing techniques based on CRISPR/Cas9 have been established and applied to genome engineering. Modified gene-editing systems also make transgene-free plants more readily available. In this review, we discuss the modifications made to gene-editing systems in recent years and summarize the capabilities, deficiencies, and applications of these modified gene-editing systems. Finally, we discuss the future developmental direction and challenges of modified gene-editing systems.

## Introduction

The world’s population is increasing rapidly, so food security has been greatly challenged. With the problem of climate change, the world’s arable land is also under threat. Therefore, increasing food production per unit of cultivated land area is a crucial strategy to maintain global food security.

Improving crops is an important way of improving agricultural productivity. The traditional breeding method obtains crops with excellent agronomic traits through natural mutation and cross-breeding. This method has been widely used in human history, and many excellent crop resources have been obtained (Liu et al., 2020). However, cross-breeding can only utilize traits already acquired by natural mutations in the crop, limiting it at the genetic level (Witkin, 1969; He et al., 2012). Chemical or radiation mutagenesis has been widely used to expand the gene pool potential. Nevertheless, this method still requires considerable labor and time to identify and obtain favorable traits through mutation breeding. In contrast, transgenesis allows transgenic elements to be inserted into a plant genome to express or silence a target gene, generating better crop traits or more efficient herbicide resistance and is labor-saving. However, genetic modification can also cross species boundaries, favorable traits may disappear generation by generation in actual agricultural production, and the insertion of exogenous fragments makes genetically modified crops subject to government supervision and public suspicion (Raman, 2017). Therefore, new methods of molecular breeding are of great importance.

Unlike transgenesis, gene editing allows targeted gene manipulation at a specific locus, so traits do not depend on transgenic elements. This technique generates transgene-free crops with favorable traits and stable inheritance in actual agricultural production (Chiu et al., 2013). Currently, the main gene-editing technologies include zinc-finger nucleases (ZFNs), transcription activator-like effector nucleases (TALENs), and clustered regularly interspaced short palindromic repeat sequences (CRISPR) with CRISPR-associated protein 9 (Cas9) (CRISPR/Cas9) (Hou et al., 2013; Joung and Sander, 2013; Gupta and Musunuru, 2014). As the newly established gene-editing system, CRISPR/Cas9 has been verified and applied in crops including, rice, maize, wheat, tomato, and potato (Jiang et al., 2013; Brooks et al., 2014; Shan et al., 2014; Zhu et al., 2016). Classical gene-editing systems typically target the crop genome, cause double-strand breaks, and then edit at the target site during DNA repair of the crop itself. Mutations obtained in this way are generally random and negatively impact crop research and breeding. To better meet the needs of crop research and breeding, gene-editing systems have been modified in various ways. These modified gene-editing systems include Cas13a, mitoTALENs, base editors (BEs), and prime editors (PEs) and enable more accurate and reliable gene editing while expanding target locations to include RNA, mitochondrial, and chloroplast genomes (Komor et al., 2016; Pereira et al., 2018; Anzalone et al., 2019). Multi-target editing makes genome engineering possible, and several different modification or transformation strategies make transgene-free crops more efficient to obtain, where transgene-free crops have significant commercial value. In this **article**, the modification and characteristics of gene-editing systems are reviewed, and their application in crops is introduced.

## Classical Gene-Editing Systems

Presently, various gene-editing tools are employed, among which ZFNs and TALENs were developed earlier.

Zinc-finger nucleases are derived from a region containing multiple zinc finger structures that can bind to DNA and from a non-specific nuclease region. A single zinc finger recognizes three specific bases, so by combining zinc finger structures, the DNA binding domain can recognize specific sequences in multiples of three bases. TALENs are similar to ZFNs in that their DNA binding regions are composed of specific conservative repeats, each of which contains 34 amino acids, and the 12th and 13th amino acids form repeat variable di-residues (RVDs), where one RVD can only recognize specific bases (Tomas et al., 2011). After the ZFNs or TALENs specifically recognize the base sequence, the target is cleaved by the nuclease domain, and the genes are edited under the action of the plant’s own DNA repair mechanism (Radecke et al., 2010; Gupta and Musunuru, 2014). Typically, ZFNs or TALENs are often used in pairs to cause double-strand breaks (DSB).

**FIGURE 1:**
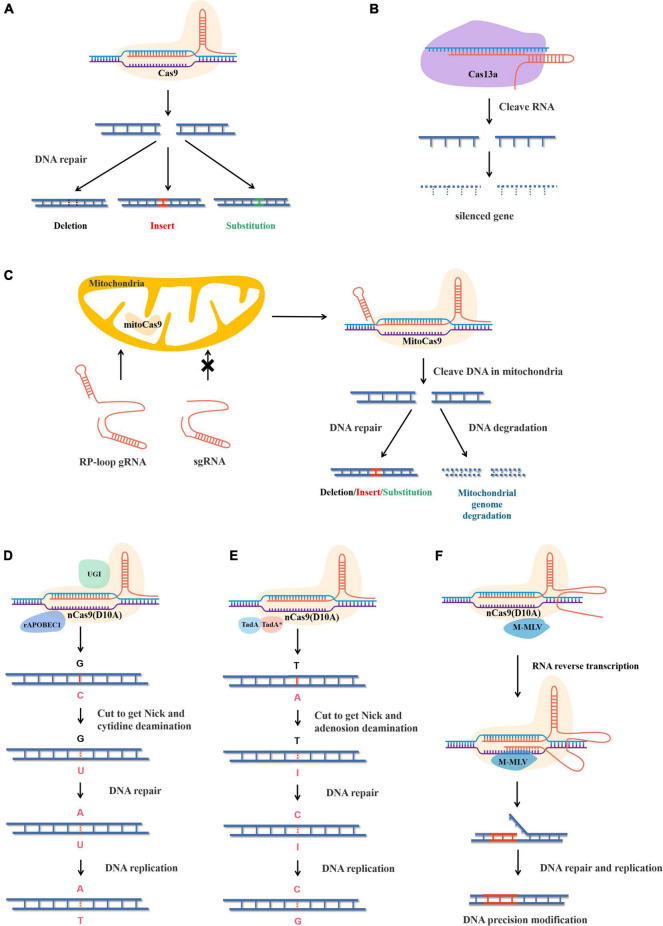
Structure and principle of different gene editing systems. **(A)** CRISPR-Cas9 cleave double-stranded DNA and causes DSB and the genes are edited under the action of the plant’s own DNA repair mechanism. **(B)** Rp-loop structure promotes sgRNA entering the mitochondrial and CRISPR-MitoCas9 result mitochondrial gene editing or genome degradation. **(C)** CRISPR-Cas13a cleaves ssRNA and causes ssRNA degradation. **(D)** The CBEs replaces bases with cytosine deaminase and T-A replace C-G under the action of plant DNA repair. rAPOBEC1, rat cytidine deaminase; GUI, uracil glycosylase inhibitor. **(E)** The adenine base editor replaces bases by adenine deaminase and G-C replace A-T under the action of plant DNA repair. TadA, wild-type *Escherichia coli* tRNA adenosine deaminase. TadA*, mutant TadA. **(F)** nCas9(D10A) cleaves single strands of DNA to form Nick, and pegRNA acts as a template to replace the sequence under the action of reverse transcriptase. Precise modification of target sequences by plant DNA repair. M-MLV, Moloney mouse leukemia virus reverse transcriptase.

Clustered regularly interspaced short palindromic repeats-CRISPR-associated protein 9 is essentially the immune mechanism of bacteria or archaea and is composed of three parts. The first is CRISPR RNA (crRNA) complementary to the targeted sequence. The second is trans-activating crRNA (tracrRNA), which binds both crRNA and Cas9. To make CRISPR-Cas9 easier to use in practical applications, CRISPR-Cas9 was modified at the molecular level. CrRNA and tracrRNA were linked by stem-loop, combining the two into a single guide RNA (sgRNA), which significantly reduced the structural complexity of the CRISPR-Cas9 gene-editing system (Jinek et al., 2012). The third part is Cas9, which contains two domains, namely the HNH domain and the RuvC-like domain. The HNH domain cuts the complementary strand of crRNA, while the RuvC-like domain cuts the opposite strand of double-stranded DNA. When CRISPR-Cas9 works, sgRNA carries the Cas9 protein to recognize the target containing a protospacer adjacent motif (PAM) NGG at the 3′ end and cleaves, resulting in DSB (Jacobs et al., 2015; Yu et al., 2016). Later, during DSB repair, the sequence is inserted by action of homologous recombination (HR), or the insertion, deletion, or alteration of bases is caused by action of non-homologous end joining (NHEJ) (Figure 1A; Bengtsson et al., 2017).

Cas12a is also used in CRISPR. The CRISPR-Cas12a (Cpf1) system uses crRNA to target sequences and has unique advantages. Firstly, Cas12a is smaller than standard Cas9, making it easier to enter the nucleus for editing. Secondly, CRISPR-Cas12a editing produces sticky ends, which is conducive to the deletion of larger fragments and the joining of foreign fragments. Thirdly, CRISPR-Cas12a can process pre-crRNA to form mature crRNA, making its multi-target editing system easier to construct and more efficient (Hu et al., 2017). Moreover, by modifying crRNA, the editing efficiency of CRISPR-Cas12a is improved, allowing it to edit genomic loci that have hitherto been difficult to edit (Hu et al., 2020). However, the PAM sequence identified by CRISPR-Cas12a is TTTV, resulting in only a 10th of the number of target sequences in plant genomes compared to CRISPR-Cas9. In addition, studies have shown that Cas12a is more sensitive to temperature than Cas9, so it needs to be modified in the many crops that grow at lower temperatures (Malzahn et al., 2019).

To date, classical gene-editing techniques have been applied in many crops, including rice, corn, wheat, potato, tomato, grape, and citrus, providing technical support for crop quality improvement, resistance to biological stress, resistance to abiotic stress, and re-domestication (Wang T. et al., 2019; Xu et al., 2019).

## Modified Gene-Editing Systems

Many modifications to the classic gene-editing systems have been made to overcome their limitations. An example is CRISPR interference systems and CRISPR activation systems, which regulate gene expression rather than edit genes. Four strategies have a high potential for modifications with gene editing capability. The first strategy is to enable gene-editing systems to be applied to RNA, mitochondrial, or chloroplast genomes. The second strategy is to change the gene-editing ability so that the gene-editing system can accurately complete the replacement of bases or the replacement and insertion of long sequences. The third strategy is multi-target editing, which would make genome engineering possible. The fourth strategy is to integrate the system with other transgenic elements or change transformation strategies to make transgene-free crops more readily available.

### Cas13: Tools for Editing RNA

In classical gene-editing systems, Cas9 and Cas12a are both type II single-component effector proteins that target DNA. In the progressive exploration of immunity in prokaryotes, the Type VI system has been elucidated. This system is characterized by the effector protein C2c2, which contains a higher eukaryotic and prokaryotic nucleotide-binding (HEPN) domain. HEPN domains are characterized by RNA enzyme activity, so C2c2 has the ability to target and edit RNA, and was named Cas13a in subsequent studies (Abudayyeh et al., 2016).

Cas13a targets sequences located 28 nt downstream of crRNA, resulting in single-stranded RNA (ssRNA) degradation and inhibiting target genes at the transcriptional level (Figure 1B). In addition, the Cas13b system has been reported to significantly improve its inhibitory efficiency compared with Cas13a in mammalian cells (Cox et al., 2017). Interestingly, cleavage of Cas13a also leads to degradation of non-target RNA *in vitro* or in prokaryotic cells, which often leads to apoptosis, but is highly specific in eukaryotic cells. In plants, Cas13a is suitable for the modification of rice, with a maximal knockdown of 78% (Abudayyeh et al., 2017).

Compared to gene silencing methods, such as RNAi or artificial microRNA, Cas13 is highly specific and, more importantly, does not depend on the plant’s own immune function. RNAi or artificial microRNA pairs dsRNA with target RNA in plants and binds with the plant Argonaute (AGO) and Dicer-like (DCL) enzymes to form an RNA-induced silencing complex (RISC), thus completing the silencing of target genes. Therefore, RNAi or artificial microRNA cannot be used to study genes related to the silencing system. Cas13 can complete the silencing of target genes only by itself, without limitations from target genes. In addition, Cas13 can also be used for RNA virus inhibition. Studies have shown that Cas13 can significantly inhibit *Tobacco mosaic virus* in tobacco leaves, so Cas13 has a high breeding potential for disease resistance in crops (Mahas et al., 2019).

Cas13 can also be modified to perform more complex functions to meet production or research needs. dCas13 was obtained by inactivating Cas13 by mutating the HEPN conserved domain and fusing dCas13 with the human adenosine deaminase acting on RNA 2 (ADAR2) deaminase domain to obtain a BE for ssRNA in mammalian cells (Cox et al., 2017). Compared with DNA editing, RNA editing is more prone to recovery and post-transcriptional regulation, which makes it highly safe and ensures its safety in the treatment of genetic diseases. However, this also makes it difficult to use in agricultural traits. dCas13can also fuse with fluorescent proteins to locate target RNA in cells, providing a new tool for RNA localization studies (Abudayyeh et al., 2017). Overall, Cas13 is more accurate than traditional silencing methods such as RNAi, but its application in crops needs further exploration.

### Gene Editing in Mitochondria and Chloroplasts

Mitochondria are responsible for plant respiration and are associated with many secondary metabolites, so they could greatly improve crop yield and quality if they can be modified (Balaban et al., 2005). Chloroplasts are directly responsible for photosynthesis and are directly related to crop yield (Chen et al., 2020). However, mitochondria and chloroplasts have relatively independent genomic and DNA repair systems and membrane systems, making traditional gene-editing methods unsuitable.

The first established method to modify mitochondria used TALENs, which were expressed in the nucleus and then transferred to the mitochondria to edit the mitochondrial genome. In rice and rape, mitoTALENs were used to precisely knock out *ORF79* and *ORF125*, which proved to be cytoplasmic male sterility genes (Kazama et al., 2019). In *Arabidopsis thaliana*, the mitoTALENs technique was used to destroy two mitochondrial genes, *AtP6-1* and *AtP6-2*, which proved that conventional mitoTALENs were more effective than single-molecule mito-compact TALENs (Arimura et al., 2020). mtZFNs were also proven useful for mitochondrial gene editing in animal cells (Gammage et al., 2018b). However, attempts to apply CRISPR/Cas9 to mitochondrial genomes encountered some difficulties, mainly because sgRNA was difficult to transport through the mitochondria membrane into the mitochondrial matrix (Gammage et al., 2018a). A recent study showed that sgRNA with short hairpin structures could be transported into the mitochondrial matrix at low levels and complete mitochondrial genome editing (Loutre et al., 2018). In a further improvement, by adding the 20-nucleotide stem-loop structure of *RNase P* to the 5′ end of sgRNA, the efficiency of sgRNA entering the mitochondrial matrix was effectively improved (Figure 1C; Hussain et al., 2021). In general, current methods exist to edit mitochondrial genomes, but they generally cause DSB, which results in long sequence changes or overall degradation during mitochondrial repair.

Although there is currently no CRISPR/Cas9-based mitochondrial BE, sgRNA with stem-loop makes it possible to establish this method. Due to difficulties encountered with sgRNA in earlier studies, new methods were developed for mitochondrial BEs. RNA-free DddA-derived cytosine base editors (DdCBEs) were produced by fusing half of the split-DddA transcription activator-like effector array protein with a uracil glycosylase inhibitor (UGI). In human mitochondria, this method can substitute C-G base pairs with A-T base pairs with high precision and efficiency (Mok et al., 2019).

Another strategy for regulating mitochondria modifies mitochondria-related RNA-binding proteins, which are often involved in mitochondrial RNA editing. In *A. thaliana*, RNA processing factor 2 (RPF2) was engineered to target *NAD6* for cleavage, virtually eliminating *NAD6* expression, leading to the accumulation and activity of complex I (Colas et al., 2018). In addition, RNA-binding proteins are involved in base conversion rather than RNA cleavage in mitochondria, providing a possibility for mitochondrial RNA base conversion (Yang et al., 2020). If the sgRNA transport method of Cas13 could be established, high-precision mitochondrial RNA editing or a base conversion system could also be established.

Compared with mitochondria, gene-editing systems in chloroplasts have developed more slowly. Although there are methods to transform chloroplasts and express sgRNA directly in chloroplasts (Ralph Bock et al., 2019), gene editing in chloroplasts is rarely reported. The first chloroplast gene editing was completed in Chlamydomonas, and the donor DNA was successfully integrated into psaA by transforming a plasmid containing the Cas9 expression cassette and a plasmid containing the donor DNA. Comparing single-target editing of intact Cas9 expression cassette samples with that of Cas9 or sgRNA deletion, single nucleotide polymorphisms in intact Cas9 expression cassette samples were not significantly increased, and no deletion mutations were found (Yoo et al., 2020). This result suggests that chloroplasts lack the NHEJ pathway, preventing the most commonly used gene-editing strategies from being implemented in chloroplasts.

Another feasible chloroplast gene-editing strategy is base editing. DdCBEs have also been applied in chloroplasts. In *A. thaliana*, ptpTALECDs were obtained by modifying DdCBEs to make them suitable for *A. thaliana*, confirming that the base editing system could efficiently edit *16S*, *rpoC1*, and *psbA* (Nakazato et al., 2021). As with mitochondria, chloroplast BEs based on CRISPR-Cas9 have not yet been developed, but based on what is available, the transformation of BEs in chloroplasts should allow base editing.

In general, mitochondrial and chloroplast editing is still in its infancy, and one of the difficulties lies in the shortcomings of the mitochondrial and chloroplast repair mechanisms. A feasible solution is to avoid DSB through BEs. However, mitochondrial and chloroplast editing has great breeding potential, especially for chloroplasts, which are the sites of photosynthesis and encode many key proteins. Rubisco, for example, is a key enzyme in the Calvin cycle. However, due to the complex biogenesis of its large subunit, the exogenous expression has been unable to produce good effects. Therefore, direct modification of Rubisco by gene editing may radically improve crop yield (Bracher et al., 2017).

### Base Editors: Precise Substitution of Bases

Clustered regularly interspaced short palindromic repeats based on NHEJ always produces random mutations with low accuracy, and HR requires donor DNA, making the system complex and inefficient (Hess et al., 2017). To overcome these limitations, researchers are working to develop editing techniques with higher efficiency, reliability, and accuracy. BEs are a newly developed precise genome-editing technique that can achieve irreversible base conversion at specific sites (Gao, 2018). After mutating Cas9 (D10A), the Cas9 nickase (nCas9) had single-strand cutting ability only. BEs are a complex consisting of the nCas9 protein, guide RNA (gRNA), and a base deaminase domain capable of converting specific base pairs (Komor et al., 2016).

Base editors are divided into two types, cytosine BEs (CBEs) and adenine BEs (ABEs). CBEs were developed first and can transform C into U. Thus, a U-G base pair can be converted into a T-A base pair during DNA repair and replication. CBEs edit bases by cytosine deaminase and contain UGIs. UGIs, derived from *Bacillus subtilis* bacteriophages, can block the activity of human uracil DNA glycosylase (UDG), preventing U from being repaired to C and reverting to a C-G base pair (Figure 1D; Molla and Yang, 2019). The earliest CBE does not contain a UGI and uses the dead Cas9 (dCas9) mutant with no cutting capability, making its C-T base-pair substitution inefficient at only 0.8–7.7% (Komor et al., 2016). In a later version, the efficiency of CBE was greatly improved by the addition of UGI and the replacement of dCas9 with nCas9 (Chen et al., 2017).

Adenine BEs replace A with I, converting I-T base pairs to C-G base pairs during DNA repair and replication. Unlike CBEs, DNA glycosylase inhibitors are not required in ABEs; however, natural adenine deaminases cannot accept DNA. With further understanding of base deaminases, the heterodimer formed by the fusion of RNA adenosine deaminase (TadA) and its mutant deoxyadenosine deaminase (TadA*, W23R, H36L, P48A, R51L, L84F, A106V, D108N, H123Y, S146C, D147Y, R152P, E155V, I156F, and K157N) was found to have a good catalytic capacity for adenine deaminase (Gaudelli et al., 2017). A highly efficient ABE was obtained by fusing this heterodimer with nCas9 (Figure 1E).

Compared with general gene editing, base editing requires higher accuracy and is often used to edit specific bases on genes. Therefore, diversification of the PAM sequence has been a hot research topic. One strategy is to look for Cas9 proteins in other species. Presently, the most widely used Cas9 is *Streptococcus pyogenes* Cas9 (spCas9), whose PAM sequence is NGG (Yu et al., 2016). However, Cas9 proteins with different PAM sequences have been found in other species through homology comparison. For example, the NmCas9 protein found in *Neisseria meningitidis* recognizes the PAM sequence NNNGMTT (Hou et al., 2013). StCas9 found in *Streptococcus thermophilus* recognizes the PAM sequence NNAGAAW (Deveau et al., 2008). However, having a longer PAM sequence does not increase the number of targets. Another strategy is to modify spCas9 through protein structure and protein-directed evolution. This strategy diversifies the PAM sequence, resulting in NG, GA, GGA, NGC, NGT, GAT, GAA, CAA, GAG, NGA, NNG, NGAG, and NGCG (Endo et al., 2018; Hu et al., 2018a,b; Ge et al., 2019; Li J. et al., 2019; Wang J. et al., 2019; Zhong et al., 2019; Qin et al., 2020; Xu et al., 2020; Zhang et al., 2020). It is worth noting that different modifications have different effects on specificity. xCas9 improves the ability of DNA specific recognition and reduces off-target efficiency, while SpRYCas9 improves off-target efficiency (Hu et al., 2018a; Walton et al., 2020). Studies in plants have shown that the editing efficiency of xCas9 on atypical PAM sequences (GAA, GAT, and GAG) was lower than for the typical PAM sequence (NGG), but Cas9-NG maintained a higher editing efficiency on the atypical PAM (NG), thus improving the BEs (Endo et al., 2018; Wang J. et al., 2019). In addition to xCas9 and Cas9-NG, the PAM sequence of the BEs was diversified to NAG, NGA, NNNRRT, NRRH, NRCH, and NRTH by mutating Cas9 in subsequent studies (Hua et al., 2019; Li et al., 2021). In the latest research, the CRISPR-SpRY Toolbox even breaks the restriction of PAM sequences and can be edited PAM-free (Ren et al., 2021). Other studies improved the editing efficiency of xCas9 through enhanced sgRNA.

Notably, shortening the PAM sequence can cause Cas9 to target its own sgRNA sequence, thus reducing editing efficiency or leading to off-target recognition, requiring optimization of the sgRNA sequence (Zhang et al., 2020). Overall, the PAM sequence diversity of BEs has been greatly enriched, vastly improving its applicability in practical production.

### Base Editors: Acquisition of Herbicide Resistance and Agronomic Traits Improvement

Presently, BEs have many applications in crops (Table 1); the most effective and promising application is the acquisition of herbicide resistance, mainly through accurate modification of acetolactate synthase (ALS), one of the key enzymes in amino acid biosynthesis. There are multiple bases in the *ALS* gene, whose substitution causes plants to acquire resistance to different herbicides (Durner et al., 1991). By CBE editing of Pro to Ser, plants acquire sulfonamide herbicide resistance, which has been widely used in crop cultivation. The strategy has been used to achieve sulfonamide herbicide resistance in wheat (*TaALS*, P174S), watermelon (*ClALS*, P190S), tomato (*SlALS1*, P186S), and potato (*StALS1*, P186S); transgene-free plants were obtained for watermelon, tomato, and potato (Shimatani et al., 2017; Tian et al., 2018; Veillet et al., 2019b). Imidazolinone herbicide resistance was achieved through the CBE mutation *AtALS* S653N much later, mainly owing to the base mutation site being outside the editing window, resulting in low editing efficiency. Finally, imidazolinone herbicide resistance plants were obtained in *A. thaliana* (Dong et al., 2020). In addition to editing *OsALS* to achieve herbicide resistance, haloxyfop-R-methyl herbicide resistance was achieved in rice by mutating C2186R in *OsACC* using the ABE7.10 system (Li et al., 2018).

**TABLE 1:** Application and improvement of BEs.

Plant species	Target gene	BE	PAM	Editing efficiency (%)	Contributions	References
*Oryza sativa*	*FTIP1e* *ALS*	Target-AID	NGG	4.3–85.7	Generation of imazamox herbicide resistance	Shimatani et al., 2017
*Oryza sativa*	*NRT1.1B* *SLR1*	APOBEC1	NGG	2.7–13.3	Regulate the conversion and utilization of nitrogen	Lu and Zhu, 2017
*Oryza sativa*	*OsCDC48* *OsNRT1.1B* *OsSPL14*	PBE	NGG	0.5–7.0	Improve editing efficiency by using nCas9	Zong et al., 2017
*Oryza sativa*	*OsPDS* *OsSBEIIb*	BE3	NGG	20	Reduced starch synthesis	Li et al., 2017
*Oryza sativa*	*OsCERK1* *OsCERK2* *OsCERK3* *ipa1* *pi-ta* *BRT1*	rBE3 rBE4	NGG NGA	10.5–38.9	Increase PAM sequence diversity	Ren et al., 2017
*Oryza sativa*	*OsSPL14* *OsSPL16* *OsSPL17* *OsSPL18* *SLR1*	ABE-P1	NGG	4.8–61.3	ABE was improved for rice	Hua et al., 2018
*Oryza sativa*	*OsRLCK185* *OsCERK1*	rBE5	NGG	2.1–27.8	CBE is optimized for GC AC sequences	Ren et al., 2018
*Oryza sativa*	*OsMPK6* *OsMPK13* *OsMPK2* *OsMPK6* *Tms9-1*	rBE14 rBE15 rBE17 rBE18	NGG	4.30–62.26	Developed a fluorescence-tracking adenine base editor	Yan et al., 2018
*Oryza sativa*	*OsACC* *OsALS* *OsCDC48* *OsDEP1, OsNRT1.1B-T1*	pABE	NGG	5.8–59.1	Herbicide resistant rice was obtained by ABE for the first time	Li et al., 2018
*Oryza sativa*	*OsCDC48* *OsNRT1.1B-T1*	PBE	NGG	44.1–82.9	Expand edit window	Zong et al., 2018
*Oryza sativa*	*OsGL1-1* *OsNAL1*	PBE	NGG	75	Intron shearing is interfered with by BE	Li Z. et al., 2019
*Oryza sativa*	*OsNGN1*	nSpCas9-NGv1-AID nSpCas9-NGv1-AID nSpCas9-NGv1-APOBEC-UGI	NG	6.3–91.1	Increase PAM sequence diversity	Endo et al., 2018
*Oryza sativa*	*OsSPL14* *OsSPL16* *OsSPL18* *GRF4* *OsSPL17* *sTOE1* *OsIDS1* *OMTN1* *SNB* *sSPL13*	ABE-P1-5 CBE-P3 CBE-p5 pKKH-Cas9 pVQR-Cas9	NAG NGA NNNRRT	2.6–74.3	Increase PAM sequence diversity	Hua et al., 2019
*Oryza sativa*	*OsDEP1*	Cas9-NG (D10A)-PmCDA1	NG	30.4–45.0	Increase PAM sequence diversity	Zhong et al., 2019
*Oryza sativa*	*IPA1* *Pikh* *WX*	NRRH-eBE3 NRCH-eBE3 NRTH-eBE3	NRRH NRCH NRTH	2.08–79.17	Increase PAM sequence diversity	Li et al., 2021
*Oryza sativa*	*OSPDS* *OsALS* *OsDSP1*	CRISPR–SpRY	PAM-free	5.3–79.0	Break the PAM sequence constraint	Ren et al., 2021
*Triticum aestivum*	*TaLOX2*	PBE	NGG	1.5–5.2	Improve editing efficiency by using nCas10	Zong et al., 2017
*Triticum aestivum*	*TaDEP1* *TaGW2*	pABE	NGG	0.4–1.1	ABE was applied in wheat for the first time	Li et al., 2018
*Triticum aestivum*	*TaALS* *TaMTL*	A3A-PBE	NGG	16.7–22.5	Expand edit window	Zong et al., 2018
*Triticum aestivum*	*TaALS*	PBE	NGG	22–78	Improve screening efficiency through co-editing	Zhang et al., 2019
*Solanum tuberosum*	*StGBSS-T6*	A5A-PBE	NGG	6.5	Expand edit window	Zong et al., 2018
*Solanum tuberosum*	*SSI protein*	CBE	NGG	71	Reduce potato starch content	Veillet et al., 2019a
*Solanum tuberosum*	*StALS* *StALS2*	Target-AID	NGG	100	Generation of sulfonylurea herbicide resistance in transegene-free potato	Veillet et al., 2019b
*Solanum lycopersicum*	*DELLA* *ETR1*	Target-AID	NGG	26.2–53.8	Altered hormone regulation	Shimatani et al., 2017
*Solanum lycopersicum*	*SlALS1* *SlALS2*	Target-AID	NGG	71.4	Generation of sulfonylurea herbicide resistance in transegene-free tomato	Veillet et al., 2019b
*Arabidopsis thaliana*	*AtALS*	BE3	NGG	1.7–7.6	The edited plants were obtained by subculture	Chen et al., 2017
*Arabidopsis thaliana*	*AtALS* *AtPDS* *AtFT* *AtLFY*	BE6.3 BE7.8 BE7.9 BE7.10	NGG	0.3–10	ABE has been verified in plants	Kang et al., 2018
*Arabidopsis thaliana*	*eIF4E*	CBE	NGG	67.9	Enhanced arabidopsis resistance to disease	Bastet et al., 2019
*Arabidopsis thaliana*	*AtMTA*	PBE	NGG	49.1	Intron shearing is interfered with by BE	Li Z. et al., 2019
*Arabidopsis thaliana*	*AtALS*	CBE	NGG	14.3–66.7	Generation of imidazolinone herbicide resistant	Dong et al., 2020
*Zea mays*	*ZmCENH3*	PBE	NGG	0.3–3.7	Improve editing efficiency by using nCas11	Zong et al., 2017
*Citrullus lanatus*	*ClALS*	BE3	NGG	22.6	Transgene-free tribenuron herbicide resistant watermelon was obtained by CBE in watermelon	Tian et al., 2018
*Brassica napus*	*BnALS* *BnPDS*	BE6.3 BE7.8 BE7.9 BE7.10	NGG	0.6–8.8	ABE has been verified in plants	Kang et al., 2018

Several agronomic key traits have also been improved by BEs. For example, the successful application of CBE to edit eIF4E in *A. thaliana* has greatly improved the plant’s resistance to disease (Bastet et al., 2019). *OsSBEIIb* and *SSI* were edited in rice and potato, respectively, resulting in decreased starch synthesis (Li et al., 2017; Veillet et al., 2019a). While gene inactivation sometimes leads to plant death, precise replacement by base editing modifies genes rather than inactivates them, making base editing hugely advantageous for studying or improving key genes in crops. However, there are still some shortcomings. BEs, especially CBEs, have been widely used, and ABEs have good specificity, but there is evidence that early CBEs are off-target at the whole gene level, resulting in a large number of C substitutions in plant genomes (Jin et al., 2019). To reduce off-target effects, the deaminase domain in BE3 was replaced with a member of the APOBEC3 cytidine deaminase family. This modification proved to have good specificity in rice (Jin et al., 2020).

### Prime Editors: Precise Modification of DNA Sequence

Non-homologous end joining always results in random DNA repair, and base editing struggles to cover all sequence modifications, so PE was invented to modify DNA sequences precisely. PE is a versatile and precise genome-editing method that uses nCas9 fused to an engineered reverse transcriptase, while template RNA is linked to sgRNA to form prime editing guidance RNA (pegRNA), which both specifies the target site and encodes the desired editing sequence. After nCas9 cleaves the single strand of DNA, template RNA paired with the sequence near the editing site, and the pegRNA template complementary strand is resynthesized under the action of Moloney murine leukemia virus (M-MLV) to complete accurate editing (Figure 1F; Anzalone et al., 2019). To date, PE has been applied in rice, wheat, corn, and tomato (Jiang et al., 2020; Lin et al., 2020; Lu et al., 2021a). However, prime editing efficiency in plants was significantly lower than reported in human cells. Therefore, various strategies have been undertaken to optimize prime editing efficiency. Improvements to the components of PE, including optimization of pegRNA length, alteration of the engineered reverse transcriptase, and enhancement of the pegRNA promoter were performed (Jiang et al., 2020; Lin et al., 2020, 2021). Also, the same edits were made using different pegRNA on the template and antisense strand (Lin et al., 2021). These methods improved prime editing efficiency in plants. In the latest study, pairs of pegRNA were able to precisely delete 710 bp or precisely replace a sequence of 108 bp (Anzalone et al., 2021). In general, the great potential of prime editing remains to be explored.

### Multi-Target Editing: Genome Engineering

For CRISPR/Cas9 systems, multi-target editing is easily implemented with multiple sgRNAs. In plants, there are two main strategies to obtain multiple sgRNAs: one strategy is to use multiple promoters to express different sgRNAs, the other strategy is to use specific sequences (tRNA, Cys4, and HDV-HH) to separate different sgRNAs and cut them into independent sgRNAs *in vivo* (Xing et al., 2014; Xie et al., 2015; Gasparis et al., 2018). At present, multi-target editing is mainly used for simultaneous editing of multiple genes or saturation knockout of a single gene, which can sometimes lead to genomic changes, such as deletion, duplication, or inversion of fragments (Zhou et al., 2014).

Currently, gene editing mainly silences genes through early termination of the coding frameshift, but this method produces truncated proteins that, in some cases, are functional and can affect research and breeding (Qing et al., 2020). In addition, mutation of individual bases *via* gene editing sometimes fails to cause changes in the secondary structure of lncRNA, so there will be no functional changes. One way to avoid these disadvantages is to delete these long fragments so that the gene disappears completely. Although many large fragment deletion results have been reported, these cases are often random and difficult to apply in practice. A feasible approach is to design multiple targets with the expectation of achieving fragment deletion between the targets. This strategy has been successfully established in rice and soybean (Wang et al., 2017; Cai et al., 2018). Furthermore, large fragment deletion efficiency can be significantly improved using microhomology-mediated end joining in rice by designing targets near microhomologous sequences (Tan et al., 2020). However, the deletion of mature and efficient large fragments is currently only reported in crops with high editing efficiency, mainly rice and soybean, and there are few reports in crops with low editing efficiency. The large fragment deletion method still requires improvement.

Multi-target editing can also cause the inversion of large segments of chromatin. In maize, by designing six targets, three on each side, 75.5 Mb were inverted on chromosome 2, simulating the mutation that occurs in nature in maize (Schwartz et al., 2020). In addition, in rice, through the design of exquisite targets, *CP12* and *PPO1* exchanged positions through a 911 kb inversion, and a new expression box was formed between the promoter of *Ubiquitin2* and *HPPD* through a 338 kb duplication. Due to the high expression levels of original *CP12* and *Ubiquitin2* in leaves, the change of promoter increased the expression levels of *PPO1* and *HPPD* by tens of folds, which enabled rice to have sufficient herbicide resistance in field tests without adverse effects on other important agronomic traits (Lu et al., 2021b). This method confirmed a new strategy for upregulating target gene expression independent of transgenic elements.

In summary, multi-target CRISPR/Cas9 has a high potential for chromatin engineering, both to simulate natural mutations and to make knockout results more reliable. However, chromatin engineering can only be applied to crops with high editing efficiency, such as rice. Application in other crops requires optimization of editing efficiency or interference with DSB repair.

### Modified Gene-Editing Systems Help Obtain Transgene-Free Crops

Obtaining transgene-free plants can be of great value for the commercialization of crops; in addition, transgene-free plants can be more easily used for other gene function studies in scientific research. Currently, CRISPR transgenic plants are obtained using Agrobacterium-mediated transformation. The Cas9-expression cassette and sgRNA-expression cassette with resistance screening genes are transferred into the plant genome, resulting in transgenic plants expressing Cas9 and sgRNA, allowing complete editing on the genome. Thus, the majority of T0 plants contain transgenic elements. Traditionally, the acquisition of transgene-free plants relies mainly on the genetic segregation of plants after self-fertilization. If only one copy of the CRISPR transgene is inserted into the genome in the T0 plants, 25% of transgene-free plants can be obtained in the T1 generation according to Mendelian genetics. However, transgenes often result in multiple copies of insertions that may be distributed on different chromosomes, making it extremely difficult to obtain transgene-free plants by self-fertilization (Lowe et al., 2009). Crossing T0 plants with wild-type plants would effectively eliminate the transgene, but it takes an extra generation to reach the goal of obtaining transgene-free and edited plants. In general, there have been many experiments to isolate transgenic free plants from the progenies of T0 plants using conventional genetic segregation, but it is laborious and time-consuming.

**FIGURE 2:**
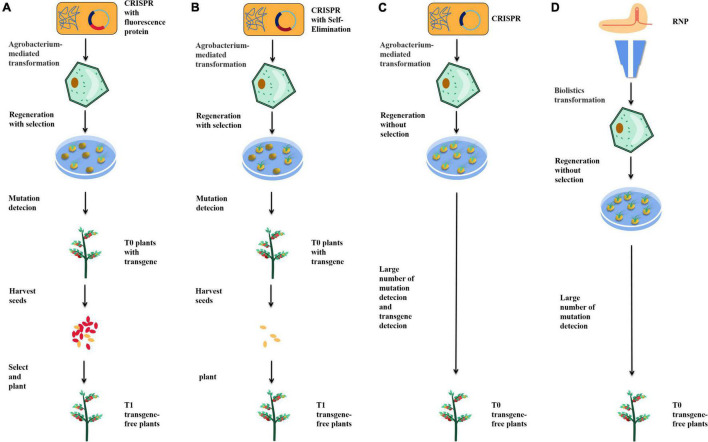
Strategies to help obtain transgene-free plants. **(A)** Fluorescence labeling screening. **(B)** Self-elimination. **(C)** Agrobacterium mediate transient transgene expression. **(D)** Bombardment-mediated RNP delivery.

In the isolation of transgene-free plants, the most time-consuming and labor-intensive work is planting, sampling, DNA extraction, and identification. Therefore, a method to identify or screen whether the seeds are genetically modified would avoid much unnecessary labor. An effective way to reduce the workload is to marker transgenic plants by fluorescence. In *A. thaliana*, by adding the mCherry fluorescent marker gene into the transgenic elements, the red fluorescence of mCherry can be used to identify and isolate transgene-free seeds (Figure 2A; Gao et al., 2016). This method has also been applied in rice, effectively reducing the amount of work required to obtain non-transgenic plants (Chang et al., 2016). In addition, GFP fluorescent protein has been used to identify transgenes. Through the 2A peptide, a picornavirus, which has been shown to have self-splitting properties in various animal cells and tissues, linking GFP to Cas9 can produce transgenic elements expressing both Cas9 and GFP without the addition of a promoter. This method can also be used to analyze the expression of Cas9 because Cas9 and GFP are expressed simultaneously by protein translation, which enables a side-analysis of editing efficiency through fluorescence intensity (Wang and Chen, 2020).

Direct transgene-free seeds can also be obtained by self-elimination. The BARNASE gene encodes a toxic protein with nuclease activity, whose expression can kill plant cells. The promoter of *REG2* was found to be specifically expressed in early rice embryos, while the rice male gametophyte-specific lethal protein CMS2 can disrupt mitochondrial function during the development of male gametophytes, leading to male sterility. By adding a REG2: *BARNASE* and 35S: *CMS2* expression cassette into the transgenic element, the transgenic male gametes and embryos were directly killed, thus ensuring that the seeds obtained were non-transgenic (Figure 2B; He et al., 2018). Compared with fluorescence screening, this method can directly obtain non-transgenic seeds without manual screening. However, the death of many seeds will lead to a decrease in the number of plant progeny, reducing the sample size obtained.

Agrobacterium mediates transient transgene expression when transgenic elements enter plant cells (Amoah et al., 2001; Krenek et al., 2015). With the help of transient transgene expression, sgRNA and Cas9 can be edited directly in plant cells without integrating the transgenic elements into the plant genome, providing a method for directly obtaining transgene-free plants in the T0 generation (Figure 2C). Using this strategy, transgene-free plants have been successfully obtained in tobacco, tomato, and potato (Chen et al., 2018; Danilo et al., 2019; Veillet et al., 2019b). The key point of this strategy is to stop using antibiotic screening after plants are edited through transient transformation so that transgene-free cells can survive. In general, 4.9–10% of transgene-free edited plants could be obtained by this method. The biggest advantage of this method lies in the direct acquisition of transgene-free edited plants in the T0 generation, without the need to separate transgenic elements in the plant progeny. However, there are four types of T0 plants: edited plants with transgenic elements, edited plants without transgenic elements, unedited plants containing transgenic elements, and wild-type plants. It is laborious to identify and isolate these four types of plants, and the number of edited plants would decrease significantly due to non-resistance screening, so this strategy remains expensive. One possible modification is to combine transient transformation with fluorescent proteins to screen non-transgenic plants during tissue culture, thereby reducing unnecessary labor.

In animal cells, the most commonly used method is to combine purified recombinant Cas9 with *in vitro* transcribed sgRNA to form cas9-sgRNA RNP complex *in vitro* and deliver the complex *in vivo* with gene gun or microinjection (Burger et al., 2016; Suzuki et al., 2018). RNP can also be used to edit plants, typically in protoplasts. Compared with plasmids, RNP does not require transgenic elements, so all the obtained plants are transgene-free (Figure 2D). Edited protoplasts have been obtained from apple and soybean using this strategy (Malnoy et al., 2016; Kim et al., 2017). However, compared with the production of edited protoplasts, the regeneration of protoplasts into plants is complicated, and many plants cannot complete this process. Methods of editing cells by bombarding immature embryos or calli with gene guns and regenerating plants by tissue culture also exist, but these methods do not enable plants to acquire resistance genes and, therefore, cannot be screened with antibiotics or herbicides. Under the condition of no selection pressure, the proportion of edited plants obtained by this method is low, so requires much effort to screen. Therefore, this method has not been widely adopted in the laboratory.

The low efficiency of RNP may be because most cells are not transformed when bombarding calli with RNP owing to the structure of calli. It is possible to improve this efficiency if a new RNP conversion method is established. In animal cells, one potential strategy is to encapsulate RNP by adenovirus proteins and use viral mechanisms to transform RNP into cells. A large amount of RNP can be wrapped in the virus, significantly improving the transformation efficiency. The efficiency of both Cas9 knockout and base editing can be significantly improved by this method (Baisong et al., 2019; Pin et al., 2019). At present, this method has not been applied in plants, and further research is needed to select and understand how to encapsulate the virus.

## Conclusion and Future Perspectives

Traditional breeding methods are no longer able to meet the food security challenges posed by population growth owing to their highly laborious and time-consuming techniques. Compared with traditional breeding, molecular breeding, especially gene-editing systems, has the advantages of being highly efficient and safe. At present, classical gene-editing systems have been successfully applied to improve disease resistance, stress resistance, and quality traits of crops (Wang T. et al., 2019). Gene-editing systems have also achieved some success in plant synthetic biology and plant microbial engineering (Gao, 2021).

However, classical gene-editing systems have their limitations. They often rely on NHEJ to make editing random, which results in poor performance when studying lethal genes (Zhang et al., 2021). Also, changes in individual bases mean that the secondary structure of RNA often does not change, making studies on plant lncRNAs rely on the deletion of large fragments, which has a very low probability. In contrast, the modified gene-editing system gives researchers more options. BEs can precisely replace bases, while PEs can precisely replace or insert sequences at target sites, which allow lethal genes to be modified rather than inactivated, thus preventing crop death. Genomic engineering can make lncRNA knockout more efficient and reliable. Cas13 has a more precise and broad range of targets that can replace traditional gene silencing techniques. Mitochondrial and chloroplast editing regulates photosynthesis and respiration, including key genes for crop yield. Optimized transgene-free access makes it easier to commercialize edited crops. In general, with the efforts of many researchers, modified gene-editing systems provide new technical means for crop research and breeding.

Like classical gene-editing systems, many modified systems are limited by species. The vast majority of systems performed well in rice but poorly in other species. On the one hand, as the main grain, rice is of high research value, so is often selected as the research target. But on the other hand, the same gene-editing system tends to be more efficient in rice and significantly less efficient in other crops, showing species-specificity. Therefore, the optimization of gene-editing systems for different species has high research value and will be an important research direction in the future. In addition to promoter and codon preference, a possible cause of the inefficiency is crop culture temperature, which is lower for most crops than rice. There is evidence that both Cas9 and Cas12a are temperature sensitive. Therefore, lifting the temperature limit may be helpful for the optimization of gene-editing systems.

The combination of different CRISPR modifications can also significantly increase the power of gene-editing systems. For example, paired pegRNAs PEs obtained by combining multi-target editing with PEs can modify longer sequences than classical PEs. BEs have excellent performance in mitochondrial and chloroplast editing owing to the defect in DSB repairability. PAM-free causes CRISPR to target itself and edit, which reduces editing efficiency and increases off-target recognition. If PAM-free is combined with Agrobacterium-mediated transient expression or bombardment-mediated RNP delivery, this deficiency may be overcome, and transgene-free plants could be obtained directly. However, compared with classical gene-editing, the editing efficiency of modified gene-editing systems will be reduced, and combinations of modified gene-editing systems may lead to lower editing efficiency. Improving transformation efficiency and screening efficiency could compensate for this issue.

Many gene-editing systems have high potential. Therefore, it is expected to see further applications of gene-editing systems in crop research and breeding in the future, which could contribute enormously to solving the problem of human food security.

## Author Contributions

GZ completed literature review and writing of this review. HZ reviewed the review and directed the revisions. Both authors contributed to the article and approved the submitted version.

## Conflict of Interest

The authors declare that the research was conducted in the absence of any commercial or financial relationships that could be construed as a potential conflict of interest.

## Publisher’s Note

All claims expressed in this article are solely those of the authors and do not necessarily represent those of their affiliated organizations, or those of the publisher, the editors and the reviewers. Any product that may be evaluated in this article, or claim that may be made by its manufacturer, is not guaranteed or endorsed by the publisher.

## References

[B1] Abudayyeh O. O. Gootenberg J. S. Essletzbichler P. Han S. Joung J. (2017). RNA targeting with CRISPR-Cas13. *Nature* 550 280–284. 10.1038/nature24049 28976959 PMC5706658

[B2] Abudayyeh O. O. Gootenberg J. S. Konermann S. Joung J. Slaymaker I. M. (2016). C2c2 is a single-component programmable RNA-guided RNA-targeting CRISPR effector. *Science* 353:f5573. 10.1126/science.aaf5573 27256883 PMC5127784

[B3] Amoah B. K. Wu H. Sparks C. Jones H. D. (2001). Factors influencing Agrobacterium-mediated transient expression of uidA in wheat inflorescence tissue. *J. Exp. Bot.* 52 1135–1142. 10.1093/jexbot/52.358.1135 11432931

[B4] Anzalone A. V. Gao X. D. Podracky C. J. Nelson A. T. Koblan L. W. (2021). Programmable deletion, replacement, integration and inversion of large DNA sequences with twin prime editing. *Nat. Biotechnol.* 2021:1133–w. 10.1038/s41587-021-01133-w 34887556 PMC9117393

[B5] Anzalone A. V. Randolph P. B. Davis J. R. Sousa A. A. Koblan L. W. (2019). Search-and-replace genome editing without double-strand breaks or donor DNA. *Nature* 576 149–157. 10.1038/s41586-019-1711-4 31634902 PMC6907074

[B6] Arimura S. I. Ayabe H. Sugaya H. Okuno M. Tamura Y. (2020). Targeted gene disruption of ATP synthases 6-1 and 6-2 in the mitochondrial genome of *Arabidopsis thaliana* by mitoTALENs. *Plant J.* 104 1459–1471. 10.1111/tpj.15041 33098708

[B7] Baisong L. Parisa J. P. Vishruti M. Farideh M. G. Mohamed S. W. (2019). Delivering SaCas9 mRNA by lentivirus-like bionanoparticles for transient expression and efficient genome editing. *Nucleic Acids Res.* 47:e44. 10.1093/nar/gkz093 30759231 PMC6486560

[B8] Balaban R. S. Nemoto S. Finkel T. (2005). Mitochondria, oxidants, and aging. *Cell* 120 483–495. 10.1016/j.cell.2005.02.001 15734681

[B9] Bastet A. Zafirov D. Giovinazzo N. Guyon-Debast A. Nogue F. (2019). Mimicking natural polymorphism in eIF4E by CRISPR-Cas9 base editing is associated with resistance to potyviruses. *Plant Biotechnol. J.* 17 1736–1750. 10.1111/pbi.13096 30784179 PMC6686125

[B10] Bengtsson N. E. Hall J. K. Odom G. L. Phelps M. P. Andrus C. R. (2017). Muscle-specific CRISPR/Cas9 dystrophin gene editing ameliorates pathophysiology in a mouse model for Duchenne muscular dystrophy. *Nat. Commun.* 8:14454. 10.1038/ncomms14454 28195574 PMC5316861

[B11] Bracher A. Whitney S. M. Hartl F. U. Hayer-Hartl M. (2017). Biogenesis and metabolic maintenance of rubisco. *Annu. Rev. Plant Biol.* 68 29–60. 10.1146/annurev-arplant-043015-111633 28125284

[B12] Brooks C. Nekrasov V. Lippman Z. B. Van Eck J. (2014). Efficient gene editing in tomato in the first generation using the clustered regularly interspaced short palindromic repeats/CRISPR-associated9 system. *Plant Physiol.* 166 1292–1297. 10.1104/pp.114.247577 25225186 PMC4226363

[B13] Burger A. Lindsay H. Felker A. Hess C. Anders C. (2016). Maximizing mutagenesis with solubilized CRISPR-Cas9 ribonucleoprotein complexes. *Development* 143 2025–2037. 10.1242/dev.134809 27130213

[B14] Cai Y. Chen L. Sun S. Wu C. Yao W. (2018). CRISPR/Cas9-Mediated deletion of large genomic fragments in soybean. *Int. J. Mol. Sci.* 19:ijms19123835. 10.3390/ijms19123835 30513774 PMC6321276

[B15] Chang Z. Chen Z. Wang N. Xie G. Lu J. (2016). Construction of a male sterility system for hybrid rice breeding and seed production using a nuclear male sterility gene. *Proc. Natl. Acad. Sci. U S A.* 2016:14145. 10.1073/pnas.1613792113 PMC5150371 27864513

[B16] Chen J. H. Chen S. T. He N. Y. Wang Q. L. Zhao Y. (2020). Nuclear-encoded synthesis of the D1 subunit of photosystem II increases photosynthetic efficiency and crop yield. *Nat. Plants* 6 570–580. 10.1038/s41477-020-0629-z 32313138

[B17] Chen L. Li W. Katin-Grazzini L. Ding J. Gu X. (2018). A method for the production and expedient screening of CRISPR/Cas9-mediated non-transgenic mutant plants. *Horticult. Res.* 5:13. 10.1038/s41438-018-0023-4 29531752 PMC5834642

[B18] Chen Y. Wang Z. Ni H. Xu Y. Chen Q. (2017). CRISPR/Cas9-mediated base-editing system efficiently generates gain-of-function mutations in Arabidopsis. *Sci. China Life Sci.* 60 520–523. 10.1007/s11427-017-9021-5 28303459

[B19] Chiu H. Schwartz H. T. Antoshechkin I. Sternberg P. W. (2013). Transgene-free genome editing in Caenorhabditis elegans using CRISPR-Cas. *Genetics* 195 1167–1171. 10.1534/genetics.113.155879 23979577 PMC3813845

[B20] Colas D. F. C. Vincis P. S. L. Small I. (2018). Targeted cleavage of nad6 mRNA induced by a modified pentatricopeptide repeat protein in plant mitochondria. *Commun. Biol.* 1:166. 10.1038/s42003-018-0166-8 30320233 PMC6181959

[B21] Cox D. Gootenberg J. S. Abudayyeh O. O. Franklin B. Kellner M. J. (2017). RNA editing with CRISPR-Cas13. *Science* 358 1019–1027. 29070703 10.1126/science.aaq0180 PMC5793859

[B22] Danilo B. Perrot L. Mara K. Botton E. Nogué F. (2019). Efficient and transgene-free gene targeting using Agrobacterium -mediated delivery of the CRISPR/Cas9 system in tomato. *Plant Cell Rep.* 38 459–462. 10.1007/s00299-019-02373-6 30649572

[B23] Deveau H. Barrangou R. Garneau J. E. Labonte J. Fremaux C. (2008). Phage Response to CRISPR-Encoded Resistance in Streptococcus thermophilus. *J. Bacteriol.* 190 1390–1400. 10.1128/JB.01412-07 18065545 PMC2238228

[B24] Dong H. Wang D. Bai Z. Yuan Y. Yang W. (2020). Generation of imidazolinone herbicide resistant trait in Arabidopsis. *PLoS One* 15:e233503. 10.1371/journal.pone.0233503 32442184 PMC7244175

[B25] Durner J. Gailus V. Böger P. (1991). New aspects on inhibition of plant acetolactate synthase by chlorsulfuron and imazaquin. *Plant Physiol.* 95 1144–1149. 10.1104/pp.95.4.1144 16668103 PMC1077664

[B26] Endo M. Mikami M. Endo A. Kaya H. Itoh T. (2018). Genome editing in plants by engineered CRISPR-Cas9 recognizing NG PAM. *Nat. Plants* 5 14–17. 10.1038/s41477-018-0321-8 30531939

[B27] Gammage P. A. Viscomi C. Simard M. L. Costa A. Gaude E. (2018b). Genome editing in mitochondria corrects a pathogenic mtDNA mutation *in vivo*. *Nat. Med.* 24 1691–1695. 10.1038/s41591-018-0165-9 30250142 PMC6225988

[B28] Gammage P. A. Moraes C. T. Minczuk M. (2018a). *Mitochondrial genome engineering: The revolution may not be CRISPR-Ized.* Amsterdam: Elsevier. 10.1016/j.tig.2017.11.001 PMC5783712 29179920

[B29] Gao C. (2018). The future of CRISPR technologies in agriculture. *Nat. Rev. Mol. Cell Biol.* 19 275–276. 10.1038/nrm.2018.2 29382940

[B30] Gao C. (2021). Genome engineering for crop improvement and future agriculture. *Cell* 184 1621–1635. 10.1016/j.cell.2021.01.005 33581057

[B31] Gao X. Chen J. Dai X. Zhang D. Zhao Y. (2016). An effective strategy for reliably isolating heritable and Cas9-free Arabidopsis mutants generated by CRISPR/Cas9-mediated genome editing. *Plant Physiol.* 2016:1794. 10.1104/pp.16.00663 27208253 PMC4936589

[B32] Gasparis S. Kala M. Przyborowski M. Lyznik L. A. Orczyk W. (2018). A simple and efficient CRISPR/Cas9 platform for induction of single and multiple, heritable mutations in barley (Hordeum vulgare L.). *Plant Methods* 14:111. 10.1186/s13007-018-0382-8 30568723 PMC6297969

[B33] Gaudelli N. M. Komor A. C. Rees H. A. Packer M. S. Badran A. H. (2017). Programmable base editing of a⋅T to G⋅C in genomic DNA without DNA cleavage. *Nature* 551 464–471. 10.1038/nature24644 29160308 PMC5726555

[B34] Ge Z. Zheng L. Zhao Y. Jiang J. Zhang E. J. (2019). Engineered xCas9 and SpCas9-NG variants broaden PAM recognition sites to generate mutations in Arabidopsis plants. *Plant Biotechnol. J.* 17 1865–1867. 10.1111/pbi.13148 31070861 PMC6737014

[B35] Gupta R. M. Musunuru K. (2014). Expanding the genetic editing tool kit: ZFNs, TALENs, and CRISPR-Cas9. *J. Clin. Investigat.* 124 4154–4161. 10.1172/JCI72992 25271723 PMC4191047

[B36] He K. Q. Hu N. B. Cui G. R. Zhou Y. L. (2012). Screening of induction medium and NaN3 mutation condition for axillary bud from stem segments of Stevia rebaudiana and POD isoenzyme analysis of mutation plantlets. *J. Plant Resour. Environ.* 21 74–79.

[B37] He Y. Zhu M. Wang L. Wu J. Wang Q. (2018). Programmed self-elimination of the CRISPR/Cas9 construct greatly accelerates the isolation of edited and transgene-free rice plants. *Mol. Plant* 11:S1196517067. 10.1016/j.molp.2018.05.005 29857174

[B38] Hess G. T. Tycko J. Yao D. Bassik M. C. (2017). Methods and applications of CRISPR-Mediated base editing in eukaryotic genomes. *Mol. Cell* 68 26–43. 10.1016/j.molcel.2017.09.029 28985508 PMC5997582

[B39] Hou Z. Zhang Y. Propson N. E. Howden S. E. Chu L. F. (2013). Efficient genome engineering in human pluripotent stem cells using Cas9 from *Neisseria meningitidis*. *Proc. Nat. Acad. Sci.* 110 15644–15649. 10.1073/pnas.1313587110 23940360 PMC3785731

[B40] Hu J. H. Miller S. M. Geurts M. H. Tang W. Chen L. (2018a). Evolved Cas9 variants with broad PAM compatibility and high DNA specificity. *Nature* 556 57–63. 10.1038/nature26155 29512652 PMC5951633

[B41] Hu X. Meng X. Li J. Wang K. Yu H. (2020). Improving the efficiency of the CRISPR-Cas12a system with tRNA-crRNA arrays. *Crop J.* 8 403–407. 10.1016/j.cj.2019.06.007

[B42] Hu X. Meng X. Liu Q. Li J. Wang K. (2018b). Increasing the efficiency of CRISPR-Cas9-VQR precise genome editing in rice. *Plant Biotechnol. J.* 16 292–297. 10.1111/pbi.12771 28605576 PMC5785341

[B43] Hu X. Wang C. Liu Q. Fu Y. Wang K. (2017). Targeted mutagenesis in rice using CRISPR-Cpf1 system. *J. Genet. Genomics* 44 71–73. 10.1016/j.jgg.2016.12.001 28043782

[B44] Hua K. Tao X. Zhu J. (2019). Expanding the base editing scope in rice by using Cas9 variants. *Plant Biotechnol. J.* 17 499–504. 10.1111/pbi.12993 30051586 PMC6335069

[B45] Hua K. Tao X. Yuan F. Wang D. Zhu J. (2018). Precise a⋅t to g⋅c base editing in the rice genome. *Mol. Plant* 11 627–630. 10.1016/j.molp.2018.02.007 29476916

[B46] Hussain S. A. Yalvac M. E. Khoo B. Eckardt S. McLaughlin K. J. (2021). Adapting CRISPR/Cas9 system for targeting mitochondrial genome. *Front. Genet.* 12:627050. 10.3389/fgene.2021.627050 33889176 PMC8055930

[B47] Jacobs T. B. LaFayette P. R. Schmitz R. J. Parrott W. A. (2015). Targeted genome modifications in soybean with CRISPR/Cas9. *BMC Biotechnol.* 15:16. 10.1186/s12896-015-0131-2 25879861 PMC4365529

[B48] Jiang W. Zhou H. Bi H. Michael F. Bing Y. (2013). Demonstration of CRISPR/Cas9/sgRNA-mediated targeted gene modification in Arabidopsis, tobacco, sorghum and rice. *Nucleic Acids Res.* 41:e188. 10.1093/nar/gkt780 23999092 PMC3814374

[B49] Jiang Y. Y. Chai Y. P. Lu M. H. Han X. L. Lin Q. (2020). Prime editing efficiently generates W542L and S621I double mutations in two *ALS* genes in maize. *Genome Biol.* 21:257. 10.1186/s13059-020-02170-5 33023639 PMC7541250

[B50] Jin S. Fei H. Zhu Z. Luo Y. Liu J. (2020). Rationally designed APOBEC3B cytosine base editors with improved specificity. *Mol. Cell* 79 728–740. 10.1016/j.molcel.2020.07.005 32721385

[B51] Jin S. Zong Y. Gao Q. Zhu Z. Wang Y. (2019). Cytosine, but not adenine, base editors induce genome-wide off-target mutations in rice. *Science* 364 292–295. 10.1126/science.aaw7166 30819931

[B52] Jinek M. Chylinski K. Fonfara I. Hauer M. Doudna J. A. (2012). A programmable dual-RNA-guided DNA endonuclease in adaptive bacterial immunity. *Science* 337 816–821. 10.1126/science.1225829 22745249 PMC6286148

[B53] Joung J. K. Sander J. D. (2013). TALENs: A widely applicable technology for targeted genome editing. *Nat. Rev. Mol. Cell Biol.* 14 49–55. 10.1038/nrm3486 23169466 PMC3547402

[B54] Kang B. Yun J. Kim S. Shin Y. Ryu J. (2018). Precision genome engineering through adenine base editing in plants. *Nat. Plants* 4 427–431. 10.1038/s41477-018-0178-x 29867128

[B55] Kazama T. Okuno M. Watari Y. Yanase S. Arimura S. I. (2019). Curing cytoplasmic male sterility via TALEN-mediated mitochondrial genome editing. *Nat. Plants* 5 722–730. 10.1038/s41477-019-0459-z 31285556

[B56] Kim H. Kim S. T. Ryu J. Kang B. C. Kim J. S. (2017). CRISPR/Cpf1-mediated DNA-free plant genome editing. *Nat. Commun.* 8:14406. 10.1038/ncomms14406 28205546 PMC5316869

[B57] Komor A. C. Kim Y. B. Packer M. S. Zuris J. A. Liu D. R. (2016). Programmable editing of a target base in genomic DNA without double-stranded DNA cleavage. *Nature* 533 420–424. 10.1038/nature17946 27096365 PMC4873371

[B58] Krenek P. Samajova O. Luptovciak I. Doskocilova A. Komis G. (2015). Transient plant transformation mediated by *Agrobacterium tumefaciens*: Principles, methods and applications. *Biotechnol. Adv.* 33 1024–1042. 10.1016/j.biotechadv.2015.03.012 25819757

[B59] Li C. Zong Y. Wang Y. Jin S. Zhang D. (2018). Expanded base editing in rice and wheat using a Cas9-adenosine deaminase fusion. *Genome Biol.* 19:59. 10.1186/s13059-018-1443-z 29807545 PMC5972399

[B60] Li J. Luo J. Xu M. Li S. Zhang J. (2019). Plant genome editing using xCas9 with expanded PAM compatibility. *J. Genet. Genomics* 46 277–280. 10.1016/j.jgg.2019.03.004 31054950

[B61] Li J. Sun Y. Du J. Zhao Y. Xia L. (2017). Generation of targeted point mutations in rice by a modified CRISPR/Cas9 system. *Mol. Plant* 10 526–529. 10.1016/j.molp.2016.12.001 27940306

[B62] Li J. Xu R. Qin R. Liu X. Kong F. (2021). Genome editing mediated by SpCas9 variants with broad non-canonical PAM compatibility in plants. *Mol. Plant* 14 352–360. 10.1016/j.molp.2020.12.017 33383203

[B63] Li Z. Xiong X. Wang F. Liang J. Li J. F. (2019). Gene disruption through base editing-induced messenger RNA missplicing in plants. *New Phytol.* 222 1139–1148. 10.1111/nph.15647 30565255

[B64] Lin Q. Jin S. Zong Y. Yu H. Zhu Z. (2021). High-efficiency prime editing with optimized, paired pegRNAs in plants. *Nat. Biotechnol.* 39 923–927. 10.1038/s41587-021-00868-w 33767395

[B65] Lin Q. Zong Y. Xue C. Wang S. Jin S. (2020). Prime genome editing in rice and wheat. *Nat. Biotechnol.* 38 582–585. 10.1038/s41587-020-0455-x 32393904

[B66] Liu Q. Yang X. Tzin V. Peng Y. Romeis J. (2020). Plant breeding involving genetic engineering does not result in unacceptable unintended effects in rice relative to conventional cross-breeding. *Plant J. Cell Mol. Biol.* 103 2236–2249. 10.1111/tpj.14895 32593184 PMC7540705

[B67] Loutre R. Heckel A. M. Smirnova A. Entelis N. Tarassov I. (2018). Can mitochondrial DNA be CRISPRized: Pro and contra. *Iubmb Life* 70 1233–1239. 10.1002/iub.1919 30184317

[B68] Lowe B. A. Prakash N. S. Way M. Mann M. T. Spencer T. M. (2009). Enhanced single copy integration events in corn via particle bombardment using low quantities of DNA. *Transgenic Res.* 18 831–840. 10.1007/s11248-009-9265-0 19381853

[B69] Lu Y. Zhu J. (2017). Precise editing of a target base in the rice genome using a modified CRISPR/Cas9 system. *Mol. Plant* 10 523–525. 10.1016/j.molp.2016.11.013 27932049

[B70] Lu Y. Tian Y. Shen R. Yao Q. Zhong D. (2021a). Precise genome modification in tomato using an improved prime editing system. *Plant Biotechnol. J.* 19 415–417. 10.1111/pbi.13497 33091225 PMC7955883

[B71] Lu Y. Wang J. Chen B. Mo S. Lian L. (2021b). A donor-DNA-free CRISPR/Cas-based approach to gene knock-up in rice. *Nat. Plants* 7 1445–1452. 10.1038/s41477-021-01019-4 34782773

[B72] Mahas A. Aman R. Mahfouz M. (2019). CRISPR-Cas13d mediates robust RNA virus interference in plants. *Genome Biol.* 20:263. 10.1186/s13059-019-1881-2 31791381 PMC6886189

[B73] Malnoy M. Viola R. Jung M. H. Koo O. Kanchiswamy C. N. (2016). DNA-Free genetically edited grapevine and apple protoplast using CRISPR/Cas9 ribonucleoproteins. *Front. Plant Sci.* 7:1904. 10.3389/fpls.2016.01904 28066464 PMC5170842

[B74] Malzahn A. A. Tang X. Lee K. Ren Q. Sretenovic S. (2019). Application of CRISPR-Cas12a temperature sensitivity for improved genome editing in rice, maize, and Arabidopsis. *BMC Biol.* 17:9. 10.1186/s12915-019-0629-5 30704461 PMC6357469

[B75] Mok B. Y. Moraes M. Zeng J. Bosch D. E. Liu D. R. (2019). A bacterial cytidine deaminase toxin enables CRISPR-free mitochondrial base editing. *Nature* 583 631–637. 10.1038/s41586-020-2477-4 32641830 PMC7381381

[B76] Molla K. A. Yang Y. (2019). CRISPR/Cas-Mediated base editing: Technical considerations and practical applications. *Trends Biotechnol.* 37 1121–1142. 10.1016/j.tibtech.2019.03.008 30995964

[B77] Nakazato I. Okuno M. Yamamoto H. Tamura Y. Itoh T. (2021). Targeted base editing in the plastid genome of *Arabidopsis thaliana*. *Nat. Plants* 7 906–913. 10.1038/s41477-021-00954-6 34211131 PMC8289735

[B78] Pereira C. V. Bacman S. R. Tania A. Ugne Z. Williams S. L. (2018). MitoTev-TALE: A monomeric DNA editing enzyme to reduce mutant mitochondrial DNA levels. *EMBO Mol. Med.* 10:e8084. 10.15252/emmm.201708084 30012581 PMC6127889

[B79] Pin L. Parisa J. P. Anthony A. Lu B. (2019). Delivering Cas9/sgRNA ribonucleoprotein (RNP) by lentiviral capsid-based bionanoparticles for efficient ‘hit-and-run’ genome editing. *Nuclc. Acids Res.* 47:e99. 10.1093/nar/gkz605 31299082 PMC6753487

[B80] Qin R. Li J. Liu X. Xu R. Yang J. (2020). SpCas9-NG self-targets the sgRNA sequence in plant genome editing. *Nat. Plants* 6 197–201. 10.1038/s41477-020-0603-9 32094641

[B81] Qing R. Tao F. Chatterjee P. Yang G. Han Q. (2020). Non-full-length Water-Soluble CXCR4(QTY) and CCR5(QTY) chemokine receptors: Implication for overlooked truncated but functional membrane receptors. *IScience* 23:101670. 10.1016/j.isci.2020.101670 33376963 PMC7756140

[B82] Radecke S. Radecke F. Cathomen T. Schwarz K. (2010). Zinc-finger nuclease-induced gene repair with oligodeoxynucleotides: Wanted and unwanted target locus modifications. *Mol. Therapy* 18 743–753. 10.1038/mt.2009.304 20068556 PMC2862519

[B83] Ralph Bock R. Forner J. Hasse C. Kroop X. Seeger S. (2019). High-efficiency generation of fertile transplastomic Arabidopsis plants. *Nat. Plants* 5 282–289. 10.1038/s41477-019-0359-2 30778165 PMC6420123

[B84] Raman R. (2017). The impact of Genetically Modified (GM) crops in modern agriculture: A review. *GM Crops Food* 8 195–208. 10.1080/21645698.2017.1413522 29235937 PMC5790416

[B85] Ren B. Yan F. Kuang Y. Li N. Zhang D. (2017). A CRISPR/Cas9 toolkit for efficient targeted base editing to induce genetic variations in rice. *Sci. China Life Sci.* 60 516–519. 10.1007/s11427-016-0406-x 28260228

[B86] Ren B. Yan F. Kuang Y. Li N. Zhang D. (2018). Improved base editor for efficiently inducing genetic variations in rice with CRISPR/Cas9-Guided hyperactive hAID mutant. *Mol. Plant* 11 623–626. 10.1016/j.molp.2018.01.005 29382569

[B87] Ren Q. Sretenovic S. Liu S. Tang X. Huang L. (2021). PAM-less plant genome editing using a CRISPR-SpRY toolbox. *Nat. Plants* 7 25–33. 10.1038/s41477-020-00827-4 33398158

[B88] Schwartz C. Lenderts B. Feigenbutz L. Barone P. Llaca V. (2020). CRISPR-Cas9-mediated 75.5-Mb inversion in maize. *Nat. Plants* 6 1427–1431. 10.1038/s41477-020-00817-6 33299151

[B89] Shan Q. Wang Y. Li J. Gao C. (2014). Genome editing in rice and wheat using the CRISPR/Cas system. *Nat. Protoc.* 9 2395–2410. 10.1038/nprot.2014.157 25232936

[B90] Shimatani Z. Kashojiya S. Takayama M. Terada R. Arazoe T. (2017). Targeted base editing in rice and tomato using a CRISPR-Cas9 cytidine deaminase fusion. *Nat. Biotechnol.* 5 441–443. 10.1038/nbt.3833 28346401

[B91] Suzuki M. Hayashi T. Inoue T. Agata K. Hirayama M. (2018). Cas9 ribonucleoprotein complex allows direct and rapid analysis of coding and noncoding regions of target genes in Pleurodeles waltl development and regeneration. *Dev. Biol.* 443 127–136. 10.1016/j.ydbio.2018.09.008 30213538

[B92] Tan J. Zhao Y. Wang B. Hao Y. Wang Y. (2020). Efficient CRISPR/Cas9-based plant genomic fragment deletions by microhomology-mediated end joining. *Plant Biotechnol. J.* 18 2161–2163. 10.1111/pbi.13390 32336015 PMC7589371

[B93] Tian S. Jiang L. Cui X. Zhang J. Guo S. (2018). Engineering herbicide-resistant watermelon variety through CRISPR/Cas9-mediated base-editing. *Plant Cell Rep.* 37 1353–1356. 10.1007/s00299-018-2299-0 29797048

[B94] Tomas C. Doyle E. L. Michelle C. Li W. Yong Z. (2011). Efficient design and assembly of custom TALEN and other TAL effector-based constructs for DNA targeting. *Nucleic Acids Res.* 39:e82. 10.1093/nar/gkr218 21493687 PMC3130291

[B95] Veillet F. Perrot L. Chauvin L. Kermarrec M. P. Guyon-Debast A. (2019b). Transgene-Free genome editing in tomato and potato plants using Agrobacterium-Mediated delivery of a CRISPR/Cas9 cytidine base editor. *Int. J. Mol. Sci.* 20:402. 10.3390/ijms20020402 30669298 PMC6358797

[B96] Veillet F. Chauvin L. Kermarrec M. P. Sevestre F. Merrer M. (2019a). The Solanum tuberosum GBSSI gene: A target for assessing gene and base editing in tetraploid potato. *Plant Cell Rep.* 38 1065–1080. 10.1007/s00299-019-02426-w 31101972

[B97] Walton R. T. Christie K. A. Whittaker M. N. Kleinstiver B. P. (2020). Unconstrained genome targeting with near-PAMless engineered CRISPR-Cas9 variants. *Science* 368 290–296. 10.1126/science.aba8853 32217751 PMC7297043

[B98] Wang J. Chen H. (2020). A novel CRISPR/Cas9 system for efficiently generating Cas9-free multiplex mutants in Arabidopsis. *ABIOTECH* 1 6–14. 10.1007/s42994-019-00011-z PMC9584096 36305009

[B99] Wang J. Meng X. Hu X. Sun T. Li J. (2019). XCas9 expands the scope of genome editing with reduced efficiency in rice. *Plant Biotechnol. J.* 17 709–711. 10.1111/pbi.13053 30549238 PMC6419569

[B100] Wang T. Zhang H. Zhu H. (2019). CRISPR technology is revolutionizing the improvement of tomato and other fruit crops. *Hortic. Res.* 6:77. 10.1038/s41438-019-0159-x 31240102 PMC6570646

[B101] Wang Y. Geng L. Yuan M. Wei J. Jin C. (2017). Deletion of a target gene in Indica rice via CRISPR/Cas9. *Plant Cell Rep.* 36 1333–1343. 10.1007/s00299-017-2158-4 28584922

[B102] Witkin E. M. (1969). Ultraviolet-Induced mutation and DNA repair. *Annu. Rev. Microbiol.* 23:487. 10.1146/annurev.mi.23.100169.002415 4899079

[B103] Xie K. Minkenberg B. Yang Y. (2015). Boosting CRISPR/Cas9 multiplex editing capability with the endogenous tRNA-processing system. *Proc. Nat. Acad. Sci.* 112 3570–3575. 10.1073/pnas.1420294112 25733849 PMC4371917

[B104] Xing H. L. Dong L. Wang Z. P. Zhang H. Y. Han C. Y. (2014). A CRISPR/Cas9 toolkit for multiplex genome editing in plants. *BMC Plant Biol.* 14:327. 10.1186/s12870-014-0327-y 25432517 PMC4262988

[B105] Xu J. Hua K. Lang Z. (2019). Genome editing for horticultural crop improvement. *Hortic. Res.* 6:113. 10.1038/s41438-019-0196-5 31645967 PMC6804600

[B106] Xu Y. Meng X. Wang J. Qin B. Wang K. (2020). ScCas9 recognizes NNG protospacer adjacent motif in genome editing of rice. *Sci. China Life Sci.* 63 450–452. 10.1007/s11427-019-1630-2 31953707

[B107] Yan F. Kuang Y. Ren B. Wang J. Zhang D. (2018). Highly efficient a⋅t to g⋅c base editing by Cas9n-Guided tRNA adenosine deaminase in rice. *Mol. Plant* 11 631–634. 10.1016/j.molp.2018.02.008 29476918

[B108] Yang Y. Liu X. Wang K. Li J. Zhu G. (2020). Molecular and functional diversity of organelle RNA editing mediated by RNA recognition motif-containing protein ORRM4 in tomato. *New Phytol.* 228 570–585. 10.1111/nph.16714 32473605

[B109] Yoo B. C. Yadav N. S. Orozco E. J. Sakai H. (2020). Cas9/gRNA-mediated genome editing of yeast mitochondria and Chlamydomonas chloroplasts. *PeerJ* 8:e8362. 10.7717/peerj.8362 31934513 PMC6951285

[B110] Yu J. Zi X. Yu H. (2016). An insight into the protospacer adjacent motif of Streptococcus pyogenes Cas9 with artificially stimulated RNA-guided-Cas9 DNA cleavage flexibility. *RSC Adv.* 6 33514–33522.

[B111] Zhang C. Kang G. Liu X. Zhao S. Yuan S. (2020). Genome engineering in plant using an efficient CRISPR-xCas9 toolset with an expanded PAM compatibility. *Front. Genome Editing* 2:618385. 10.3389/fgeed.2020.618385 34713242 PMC8525348

[B112] Zhang Q. Wang Y. Xie W. Chen C. Ren D. (2021). OsMORF9 is necessary for chloroplast development and seedling survival in rice. *Plant Sci.* 307:110907. 10.1016/j.plantsci.2021.110907 33902846

[B113] Zhang R. Liu J. Chai Z. Chen S. Bai Y. (2019). Generation of herbicide tolerance traits and a new selectable marker in wheat using base editing. *Nat. Plants* 5 480–485. 10.1038/s41477-019-0405-0 30988404

[B114] Zhong Z. Sretenovic S. Ren Q. Yang L. Bao Y. (2019). Improving plant genome editing with High-Fidelity xCas9 and non-canonical PAM-Targeting Cas9-NG. *Mol. Plant* 12 1027–1036. 10.1016/j.molp.2019.03.011 30928637

[B115] Zhou H. Liu B. Weeks D. P. Spalding M. H. Yang B. (2014). Large chromosomal deletions and heritable small genetic changes induced by CRISPR/Cas9 in rice. *Nucleic Acids Res.* 42 10903–10914. 10.1093/nar/gku806 25200087 PMC4176183

[B116] Zhu J. Song N. Sun S. Yang W. Zhao H. (2016). Efficiency and inheritance of targeted mutagenesis in maize using CRISPR-Cas9. *J. Genet. Genomics* 43 25–36. 10.1016/j.jgg.2015.10.006 26842991

[B117] Zong Y. Song Q. Li C. Jin S. Zhang D. (2018). Efficient C-to-T base editing in plants using a fusion of nCas9 and human APOBEC3A. *Nat. Biotechnol.* 2018:4261. 10.1038/nbt.4261 30272679

[B118] Zong Y. Wang Y. Li C. Zhang R. Chen K. (2017). Precise base editing in rice, wheat and maize with a Cas9-cytidine deaminase fusion. *Nat. Biotechnol.* 35 438–440. 10.1038/nbt.3811 28244994

